# On Flat Foot and Its Treatment

**Published:** 1897-01-30

**Authors:** J. Lacy Firth

**Affiliations:** Assistant Surgeon, Bristol General Hospital.


### ON FLAT FOOT AND ITS TREATMENT.

By J. LACY FIRTH, M.D., M.S.(Lond.), F.R.C.S., Assistant Surgeon, Bristol General Hospital.

(*Continued from page* 282.)

The appearances of flat foot are easy to recognise. The normal arch of the inner border of the foot is lost, and may be replaced by a down and inward convexity. The sole is, therefore, flatter than normal, and touches the ground in standing and walking over a larger surface. Normally, in standing, the heel, the outer border, and anterior part of the foot beneath the heads of the metatarsals actually rest upon the ground. In flat foot the whole of the sole touches the ground.

The increase of area of contact with the ground in flat foot is easily shown by comparing the footprints of a flat and of a normal foot upon smoked paper. The inner border, in addition to being depressed, is lengthened. The outer border is shortened, and may be raised off the ground. The foot is abducted at the median tarsal, and subastragaloid joints and the heel is deflected outwards by the rotation of os calcis on its antero-posterior axis, and comes more and more in the advancing deformity to lie beneath the external malleolus. The abduction at Chopart's joint leaves the inner part of the head of the astragalus uncovered by the navicular bone, and the latter bone in the severest cases is almost completely dislocated on to the outer side of the neck of the astragalus. All the tarsal bones are rotated inwards on their antero-posterior axes. Hence the tubercle of the scaphoid projects downwards rather than inwards. Three bony prominences can be felt on the inner side of the foot, viz., of the internal malleolus, the head of the astragalus, and the tubercle of the navicular bone. All of these are lower than natural. If dots be placed over these prominences, and if they be joined by a curved line, this curve will be convex downwards, the middle dot being the lowest by reason of the greater descent of head of the astragalus. Normally such a line would be concave downwards.[1] The ball of the toe is less prominent than normal, and the metatarso-phalangeal joint prominent in the dorsum. The dorsum of the foot is flattened, and the prominence of the instep lost. The abduction of the foot at the median tarsal joint can be well demonstrated by drawing one line forwards along the middle of the heel and another backwards over the space between the first and second metatarsal bones. These lines, if produced, will intersect, forming an outwardly directed angle, the angle of abduction.[2]

The chief symptoms of flat foot are pain, weakness, alteration of gait, and swelling. The pain may be in the median tarsal joint, probably from the stretching of ligaments and abnormal pressure upon the bones. Pain is also produced over the prominent head of the astragalus and bursæ are often developed, and are due to the pressure of the soft structures between the bone and the ground. Often there is extreme pain on movement, and in the metatarso-phalangeal joint of the great toe. Walsham thinks this is much aggravated by the wearing of a boot which is too short to allow for the increased length of the inner border of the foot. Pain in the leg and even in the thigh of aching character is frequent.

The weakness of the foot may be shown by the inability of the patient to rise upon the toes, or to invert the sole when standing, or even when sitting. All elasticity of the foot is lost, and in walking it is brought flat down like an inert mass.

**Treatment.**—This depends upon the degree of deformity. In the incipient cases the patient still possesses the power of rising on tiptoe, and of inverting the sole and standing on the outer border of the foot, and the arch is restored when the feet are removed from the ground. The treatment of such cases is by rest and exercises. Rest in bed or on a couch or sitting, by removing the body-weight from the feet gives the weakened ligaments an opportunity of regaining strength. Later, or from the first in the mildest cases, the patient having been instructed to avoid long standing and weight carrying, must perform certain exercises which are calculated to strengthen the muscles which normally support the arch of the foot. If long standing is unavoidable, it should be done with the feet straight forwards (adducted), or even with the toes turned slightly inwards. Also at intervals the feet should be brought into contact with the inner borders raised and resting one against the other, and so kept. By this means rest will be given to the adductors and yet the arch will receive support. Occasionally, also, the patient should rise on tiptoe. When walking the feet should also be kept in the straightforward position, and the weight thrown as much on to the outer side of the foot as possible. But the most important treatment for flat foot, whenever the deformity and consequent weakness are slight enough to permit its adoption, is that by exercises. Ellis and Roth have laid stress upon these exercises, and devised those specially useful. They may be described as follows: (1) For five minutes three, four, or five times daily, the patient, standing erect, with head thrown back, arms by the sides, back and knees straight, the heels turned well outwards, the toes inwards, and the great toes touching in front, should rise upon tiptoe, and then immediately sink down again upon the heels rhythmically. Walsham recommends this to be done before a clock, so that the patient may rise and fall with each swing of the pendulum. It is not advisable to remain on tiptoe, as that would tend to stretch the ligaments. (2) Sitting with the knees crossed, and with the exercising leg extended at the knee and rotated in, the foot should be circumducted. This may be done for five minutes also, the foot moving for half the time in one direction and for half in the other. The surgeon may assist the movement if necessary at first, or later may resist it. This exercises all the muscles which move the foot seriation. (3) Standing as in the first exercise, the patient should rhythmically raise the inner borders of the feet and rest on their outer borders, and then sink back to the usual position. This may be done for five minutes also. Then for five minutes the patient should walk about on the outer borders of the feet. The above treatment will be sufficient to cure the slight cases. For more severe forms, during the intervals between the exercises, some appliances must be used to support the arch of the foot. In all cases a properly-made boot is essential. The boot should lace up, and the uppers should not meet, to allow for stretching. The toes must be broad and the inner border straight (or even concave inwards). It is most important to cure hallux valgus if present, or prevent it if not. If it be present the boot should be provided with a toe post to rest against the outer border of the great toe, and prevent its deflection in that direction. The socks must be broad at the toe end, and it is the best to have socks with a special digital compartment for the great toe. The sole and heel of the boot should be thicker by a quarter to half an inch on the inner border, to throw the weight of the body to a greater degree upon the outer part of the foot.

For cases of moderate severity, as we have said, the arch will require special support. Valgus pads fixed in the boot, or unfixed, and of felt, rubber, steel, and other materials have been much used. They are moulded to fit the restored arch of the foot, and in some cases give relief. Walsham describes (op. cit.) a boot he has found very useful, constructed as above, but with an outside iron extending above to a half-circlet for the back of the leg, and placed just below the head of the fibula, and fixed by straps and a buckle in front. The iron is jointed at the ankle-level. An inside T-strap supports the inner side of the foot, and its horizontal pieces buckle outside over the leg-iron. To support the instep an elastic band is fixed to the outer border of the boot inside, opposite the middle of the tarsus, and passing inwards under the arch and then upwards is fixed to the calf circlet. It may be used with or without a valgus pad. For the majority of cases a boot like the above without the special instep support will be sufficient.

In severe cases of flat foot, in which the patient cannot restore the arch even momentarily, it is best to give an anæsthetic, and by force restore the arch as completely as possible, and fix the foot for a month in plaster of Paris, with the sole well inverted. If a heel is made, and the plantar surface thickened with strips of plaster of Paris bandage on the inner side, the patient can walk in the splints. At the end of a month, the rest will probably have produced such improvement that the exercises may be practised, and one of the boots with leg irons worn.

For the severest cases of all various operations have been devised. The tendons of the peronei will often be found as a tense band passing forwards below the external malleolus, but the above treatment will overcome their resistance, and tenotomy is unnecessary.

Ogston's operation has been favourably spoken of by many authors. It is an excision of the astragalo-scaphoid joint. The details of the operation we shall not enter into. Such severe treatment is called for with extreme rarity.

[1] "Deformities of the Human Foot." (Walsham and Hughes.)
[2] Walsham and Hughes, op. cit.

